# MiR-24-3p attenuates IL-1β-induced chondrocyte injury associated with osteoarthritis by targeting BCL2L12

**DOI:** 10.1186/s13018-021-02378-6

**Published:** 2021-06-11

**Authors:** Jin Xu, Xiaozhong Qian, Ren Ding

**Affiliations:** 1 Department of Orthopedics, Baoshan District Shanghai Integrated Traditional Chinese and Western Medicine Hospital, No. 181 Youyi Road, Baoshan District, Shanghai, 201999 China; 2 Department of Orthopedics, Shuguang Hospital Baoshan Branch, Shanghai University of Traditional Chinese Medicine, Shanghai, 201999 China; 3 Department of Orthopedics, Community Health Center of Songnan Town, Baoshan District, Shanghai, 200441 China

**Keywords:** Chondrocytes, Inflammation, Osteoarthritis, miR-24-3p

## Abstract

**Background:**

MiR-24-3p has been reported to be involved in an osteoarthritis (OA)-resembling environment. However, the functional role and underlying mechanism of miR-24-3p in chondrocyte injury associated with OA remains unknown.

**Methods:**

The expression of miR-24-3p was determined using reverse transcription quantitative PCR analysis in OA cases and control patients, as well as IL-1β-stimulated chondrocyte cell line CHON-001. The cell viability was analyzed by CCK-8 assay. Apoptosis status was assessed by caspase-3 activity detection. The pro-inflammatory cytokines (TNF-α and IL-18) were determined using ELISA assay. The association between miR-24-3p and B cell leukemia 2-like 12 (BCL2L12) was confirmed by luciferase reporter assay.

**Results:**

We first observed that miR-24-3p expression level was lower in the OA cases than in the control patients and IL-1β decreased the expression of miR-24-3p in the chondrocyte CHON-001. Functionally, overexpression of miR-24-3p significantly attenuated IL-1β-induced chondrocyte injury, as reflected by increased cell viability, decreased caspase-3 activity, and pro-inflammatory cytokines (TNF-α and IL-18). Western blot analysis showed that overexpression of miR-24-3p weakened IL-1β-induced cartilage degradation, as reflected by reduction of MMP13 (Matrix Metalloproteinase-13) and ADAMTS5 (a disintegrin and metalloproteinase with thrombospondin motifs-5) protein expression, as well as markedly elevation of COL2A1 (collagen type II). Importantly, BCL2L12 was demonstrated to be a target of miR-24-3p. BCL2L12 knockdown imitated, while overexpression significantly abrogated the protective effects of miR-24-3p against IL-1β-induced chondrocyte injury.

**Conclusions:**

In conclusion, our work provides important insight into targeting miR-24-3p/BCL2L12 axis in OA therapy.

## Introduction

Osteoarthritis (OA) as a highly prevalent degenerative joint disease causes severe pain, joints stiffness, and even disability in older and middle people worldwide [1], whose primary characteristics include articular cartilage degradation caused by the imbalance of extracellular matrix (ECM) components, joint inflammation, and subchondral bone sclerosis [2, 3]. Chondrocytes, as the only cells in the healthy cartilage, play a crucial role in maintaining the balance of the extracellular matrix and tissue homeostasis [4]. Several risk factors such as pro-inflammatory cytokines and abnormal mechanical stress-induced molecular events (apoptosis, cell death, necrosis, and ECM degradation) in chondrocytes have been reported to be closely correlated with the pathological process of OA [5–7]. Therefore, gaining a better understanding on the molecular mechanisms underlying chondrocyte injury is of great significance in developing effective therapies against OA.

MicroRNAs (miRNAs/miRs) have been reported to regulate a variety of biological processes, such as cell proliferation, differentiation, and apoptosis, and miRNAs are identified as important regulators involved in the development and progression of human diseases, including OA [8, 9] by negatively modulating protein-coding gene expression via binding to the 3′-untranslated region (3′UTR) of target mRNAs [10]. Among then, miR-24-3p played important functional roles in several diseases. For example, miR-24-3p was highly expressed in tumor tissues and promoted the cell proliferation, migration, and invasion in cancer cells, including lung cancer [11], prostate cancer [12], and bladder cancer [13]. Tan et al. [14] and Xiao et al. [15] reported that miR-24-3p exerted cardioprotective effects in myocardial ischemia/reperfusion (I/R) injury. Similarly, Shen et al. [16] demonstrated that miR-24-3p may ameliorate inflammatory response and cellular apoptosis in hepatic I/R process, which might be a potential therapeutic target for preventing liver I/R development and progression. Interestingly, a recent study by Ragni et al. [17] who pointed that miR-24-3p was involved in adipose-derived mesenchymal stem cells (ASCs) regulating cell homeostasis and regenerative pathways in an OA-resembling environment. However, the involvement and underlying mechanism of miR-24-3p in chondrocyte injury associated with the pathogenesis of OA remains unknown.

B cell leukemia 2-like 12 (BCL2L12), a new member of the apoptosis-related BCL2 gene family contains a highly conserved BH2 domain, and a BH3-like motif and a proline-rich region. So far, it still exerted controversy on the role of BCL2L12 as an anti-apoptotic or pro-apoptotic factor in the control of apoptosis, which was considered to be cell type-dependent [18, 19]. In our previous investigation, BCL2L12 was identified as a potential target gene of miR-24-3p. Moreover, BCL2L12 expression level was observed to be significantly upregulated in the osteoarthritic samples contrary to the physiologically healthy samples [20]. Based on these facts, we thus speculated that miR-24-3p played a critical role in the pathogenesis of OA by regulating chondrocyte injury via targeting BCL2L12.

To validate our hypothesis, we first analyzed the expression of miR-24-3p in OA cartilage tissues and IL-1β-stimulated human chondrocyte cell line CHON-001. We next tested the impacts of miR-24-3p overexpression on cell viability, apoptosis, inflammation, and cartilage ECM degradation in the in vitro cultured IL-1β-induced OA chondrocyte. Moreover, we explored the association between miR-24-3p and BCL2L12 in IL-1β-induced OA chondrocyte.

## Materials and methods

### Knee tissue collection

Human cartilage specimens were collected after total knee arthroplasty from 32 patients who were diagnosed as OA (aged 42–58 years, 22 males and 10 females) according to the American College of Rheumatology (ACR) classification criteria [21]. Meanwhile, the cartilage from 32 nonarthritic knee joints of the donors who suffered from a trauma without known history of joint disease were used as normal controls (aged 36–55 years, 21 males and 11 females). The collection of specimens were under the approval of the Ethics Committee of Baoshan District Shanghai Integrated Traditional Chinese and Western Medicine Hospital (Shanghai, China). Informed consent was signed by each participant.

### Cell culture and stimulation

CHON-001, a human chondrocyte cell line derived from normal articular cartilage was purchased from American Type Culture Collection (ATCC, Manassas, VA, USA). CHON-001 cells were cultured in Dulbecco’s modified Eagle’s medium (DMEM, Gibco, Grand Island, USA) with 10% fetal bovine serum (FBS, Gibco) and 0.1% mg/ml G-418 (Gibco) at 37 °C under a humidified atmosphere containing CO_2_. The stable cultured CHON-001 cells were stimulated with 10 ng/mL IL-1β (Sigma Aldrich, St. Louis, MO, USA) for 24 h to establish OA model in vitro.

### Cell transfection

The specific miR-24-3p mimics and scramble negative control (miR-NC), small interfering RNA targeting BCL2L12 (si-BCL2L12) and its negative control (si-NC), as well as pcDNA3.1-BCL2L12 overexpression vector and pcDNA3.1 empty vector were synthesized by GenePharma Co., Ltd. (Shanghai, China). Next, CHON-001 cells at a density of 5 × 10^5^ cells/well were seeded into six-well plates and transfected with the above oligonucleotides or vectors according to the experimental requirements in accordance with the manufacturer’s instructions of lipofectamine 2000 (Invitrogen, CA, USA). Forty-eight hours after transfection, CHON-001 cells were stimulated with IL-1β (10 ng/ml) for 24 h, which were harvested for further studies.

### Reverse transcription quantitative PCR

Total RNA sample was isolated using TRIzol® reagent (Thermo Fisher Scientific, Inc.), and reverse transcription was performed with the Mir-X miRNA First-Strand Synthesis Kit or PrimeScriptTM RT reagent Kit with gDNA Eraser (TaKaRa, Dalian, China). Reverse transcription quantitative PCR was carried out on an Applied Biosystems 7300 real-time PCR system (Applied Biosystems; Thermo Fisher Scientific, Inc.) with a SYBR Premix ExTaq kit (TaKaRa) following the thermocycling conditions: Initial denaturation at 95°C for 1 min, followed by 50 cycle of 95°C for 30 s, 55°C for 45 s, and 72°C for 35 s. The sequences of primers used for PCR analysis were as follows: miR-24-3p forward: 5′-TTTGGCTCAGTTCAGCAG-3′ and reverse: 5′- TTTGGCACTAGCACATT-3′; U6 forward: 5′-CGGGTTTGTTTTGCATTTCT-3′ and reverse: 5′-AGTCCCAGCATGAACAGCTT-3′; BCL2L12 forward: 5′-GACTTCTACACCCTGGTGGC-3′ and reverse: 5′-GCCTCCTTCTCCGTGGCT-3′; GAPDH forward: 5′-CAGCCTCAAGATCATCAGCA-3′ and reverse: 5′-TGTGGTCATGAGTCCTTCCA-3′. Relative expression of miR-24-3p and BCL2L12 was calculated with the 2^−ΔΔCt^ method [22]. U6 and GAPDH were used as the internal control for miR-24-3p and BCL2L12, respectively. The experiment was performed in triplicate.

### Cell viability assay

Transfected CHON-001 cells were plated onto a 96-well plate at a density of 3 × 10^3^ cells/well and cultured for 0, 24, 48, and 72 h, respectively. At each time point, cells in each well were incuabted for 2 h with 10 μL Cell Counting Kit-8 (CCK-8) solution (Dojindo, Kumamoto, Japan) at 37 °C. The absorbance was then measured at a wavelegnth of 450 nm by a microplate reader (Bio-Rad, Hercules, USA). The experiment was performed in triplicate.

### Caspase-3 activity analysis

Apoptosis of CHON-001 cells was assessed by analyzing the caspase-3 activity in accordance with the instructions provided by commercial Caspase-3 Colorimetric Activity Assay Kit (Millipore, Billerica, MA, USA). With an ELISA reader (Bio-Rad Laboratories, Inc., Hercules, CA, USA), the absorbance at a wavelegnth of 405 nm was measured and normalized by control group. The experiment was performed in triplicate.

### Enzyme-linked immunosorbent assay (ELISA)

Inflammation status of CHON-001 cells was evaluated by determining the release of pro-inflammatory cytokines (TNF-α and IL-18) in the cellular supernants in accordance with the instructions provided by Valukine ELISA kit (R&D Systems, Inc., Minneapolis, MN, USA). The experiment was performed in triplicate.

### Target prediction and luciferase reporter assay

The target genes of miR-24-3p were predicted using TargetScan 7.1 (http://www.targetscan.org/). The predicted and corresponding mutated 3′-UTR fragments of human BCL2L12 mRNA (CUGAGCC) containing a putative miR-24-3p-binding site were cloned into pGL3 vector (Promega Corporation, Madison, WI, USA) to construct wild-type BCL2L12 (WT BCL2L12) and mutanted BCL2L12 (MUT BCL2L12) plasmid, respectively, by the GenePharma. For the luciferase reporter assay, CHON-001 cells at a density of 3 × 10^5^ cells per well were co-transfected with 20 nM miR-24-3p mimics or 20 nM miR-NC and 0.2 μg WT BCL2L12 or 0.2 μg MUT BCL2L12 using lipofectamine 2000 (Invitrogen). The cells were harvested at 48 h transfection for analysis of relative luciferase activity using the Dual-Luciferase Reporter Assay system (Promega Corporation). The experiment was performed in triplicate.

### Western blot analysis

Extraction of total protein sample was performed using ice-cold RIPA lysis buffer (Beyotime Biotechnology, Shanghai, China), and protein concentration was determined using a BCA Protein Assay Kit (Beyotime Biotechnology). After separation of protein sample (30 μg) through 10% SDS-PAGE, we tranferred the separated protein onto PVDF membranes (Millipore) and blocked them with tris-buffered saline and Tween (TBST) containing 5% skim milk for 2 h at room temperature. Then, the membarnes were incuabted overnight at 4 °C with primary antibodies against BCL2L12, MMP-13, ADAMTS-5, ACAN, COL2A1, and GAPDH (all from Abcam Cambridge, MA, USA), followed by incubated with horseradish peroxidase-conjugated secondary antibody at room temperature for 1.5 h. All the targeted protein bands were visualized using enhanced chemiluminescence detection reagents (GE healthcare Life Science, Pittsburgh, USA).

### Statistical analysis

All quantitative data were analyzed using GraphPad Prism 6.0 (GraphPad Software Lin., La Jolla, USA) and presented as mean ± standard deviation (SD) of three independent experiments. Differences between two groups were evaluated by Student’s *t* test, and differences among multiple groups were investigated by one-way analysis of variance followed by Tukey’s test, which were considered to be statistically significant when *P* value less than 0.05.

## Results

Expression level of miR-24-3p was downregulated in OA cartilage tissues and IL-1β-induced chondrocytes.

**Fig. 1:**
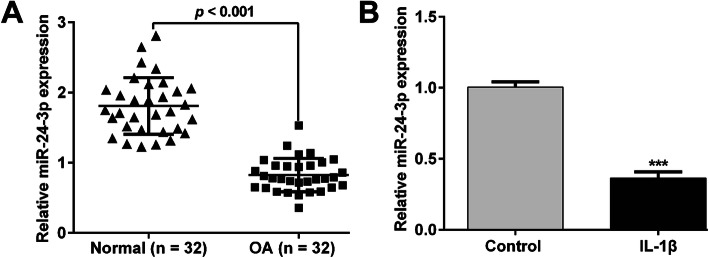
Expression of miR-24-3p in OA cartilage tissues and IL-1β-induced chondrocytes. **a** The expression of miR-24-3p in OA cartilage tissues (*n* = 32) and normal cartilage tissues (*n* = 32) was determined by reverse transcription quantitative PCR. **b** The expression of miR-24-3p in IL-1β-induced chondrocytes and normal untreated chondrocytes was detected by reverse transcription quantitative PCR. Data were presented as mean ± SD of three independent experiments. ***p < 0.001, compared with control

To confirm whether miR-24-3p was involved in the pathological process of OA, we first collected the cartilage tissues from OA patients and age-matched normal controls and determined the expression of miR-24-3p using reverse transcription quantitative PCR. As shown in Fig. 1a, the expression of miR-24-3p in patients with OA was significantly lower than that in matched normal controls. Moreover, we established the OA model in vitro using IL-1β-stimulated CHON-001 cells. Consistently, miR-24-3p expression was distinctly decreased in IL-1β-stimulated chondrocytes compared with that in untreated control group (Fig. 1b). These results indicated that miR-24-3p expression was suppressed in an OA microenvironment.

### Overexpression of miR-24-3p significantly inhibited IL-1β-induced chondrocyte injury in vitro

**Fig. 2:**
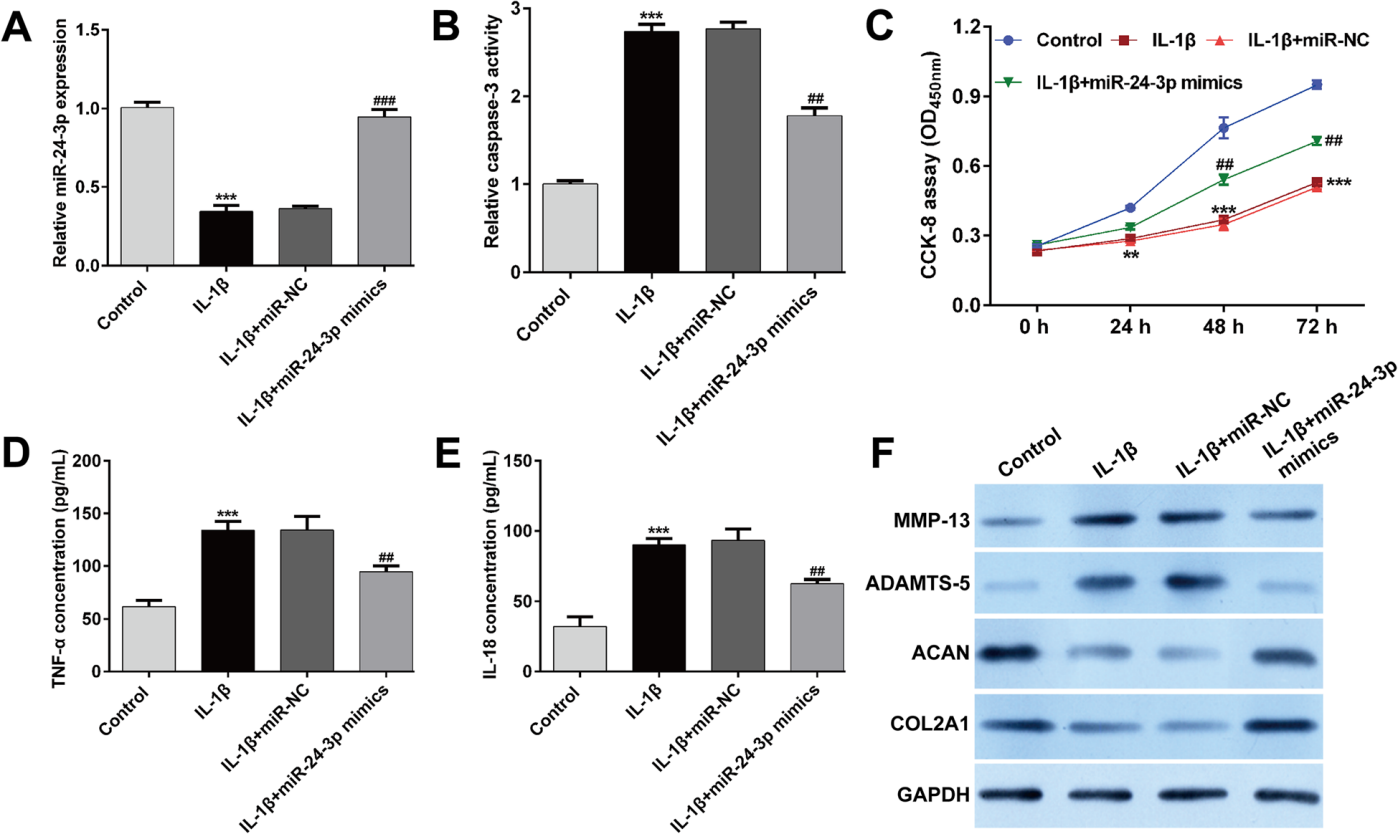
Effects of miR-24-3p overexpression on IL-1β-induced apoptosis, inflammation, and cartilage ECM degradation. Chondrocyte cell line CHON-001 was transfected with miR-24-3p mimics or miR-NC, followed by IL-1β stimulation. The untreated cells were used as control group. **a** The expression of miR-24-3p was detected by reverse transcription quantitative PCR. **b** Caspase-3 activity was analyzed using by commercial Caspase-3 Colorimetric Activity Assay Kit. **c** Cell viability was examined by CCK-8 assay. The release of TNF-α (**d**) and IL-18 (**e**) in supernatant of CHON-001 cells from different groups was determined by ELISA assay. Data were presented as mean ± SD of three independent experiments. **p < 0.01, ***p < 0.001, compared with control; ##*p* < 0.01, ###*p* < 0.001, compared with IL-1β + miR-NC; (**f**) The protein levels of MMP-13, ADAMTS-5, aggrecan (ACAN), and collagen type II (COL2A1) were measured by Western blot assay

To further investigate the functional role of miR-24-3p during the progress of OA, we manipulated the expression level of miR-24-3p in IL-1β-stimulated CHON-001 cells and tested the transfection efficiency of miR-24-3p mimics using reverse transcription quantitative PCR. As depicted in Fig. 2a, reduced miR-24-3p expression in CHON-001 cells under IL-1β stimulation was significantly elevated by transfection with miR-24-3p mimics compared with miR-NC transfection, which confirmed that miR-24-3p overexpression was successfully constructed in vitro. Subsequently, we analyzed the effect of miR-24-3p overexpression on IL-1β-induced chondrocyte injury. The results from caspase-3 activity assay (Fig. 2b) and CCK-8 assay (Fig. 2c) showed that miR-24-3p overexpression significantly reversed the increased apoptosis and decreased cell viability induced by IL-1β stimulation in CHON-001 cells. Analysis of inflammation by ELISA assay revealed that the release of TNF-α (Fig. 2d) and IL-18 (Fig. 2e) in culture supernatants was significantly elevated by IL-1β stimulation, which was attenuated after miR-24-3p mimics transfection. Furthermore, we investigated the influences of miR-24-3p on IL-1β-induced cartilage degradation by analyzing the expression of MMP-13, ADAMTS-5, COL2A1, and ACAN in IL-1β-stimulated chondrocytes. The results from western blot analysis exhibited that miR-24-3p overexpression weakened the IL-1β-induced elevation of MMP-13 and ADAMTS-5 protein expression, as well as markedly reversed the IL-1β-induced reduction of COL2A1 and ACAN protein expression in CHON-001 cells (Fig. 2f). These data demonstrated that miR-24-3p could reverse the effects of IL-1β stimulation on apoptosis, inflammation, and cartilage ECM degradation.

MiR-24-3p suppressed BCL2L12 expression by directly targeting its 3′UTR

**Fig. 3:**
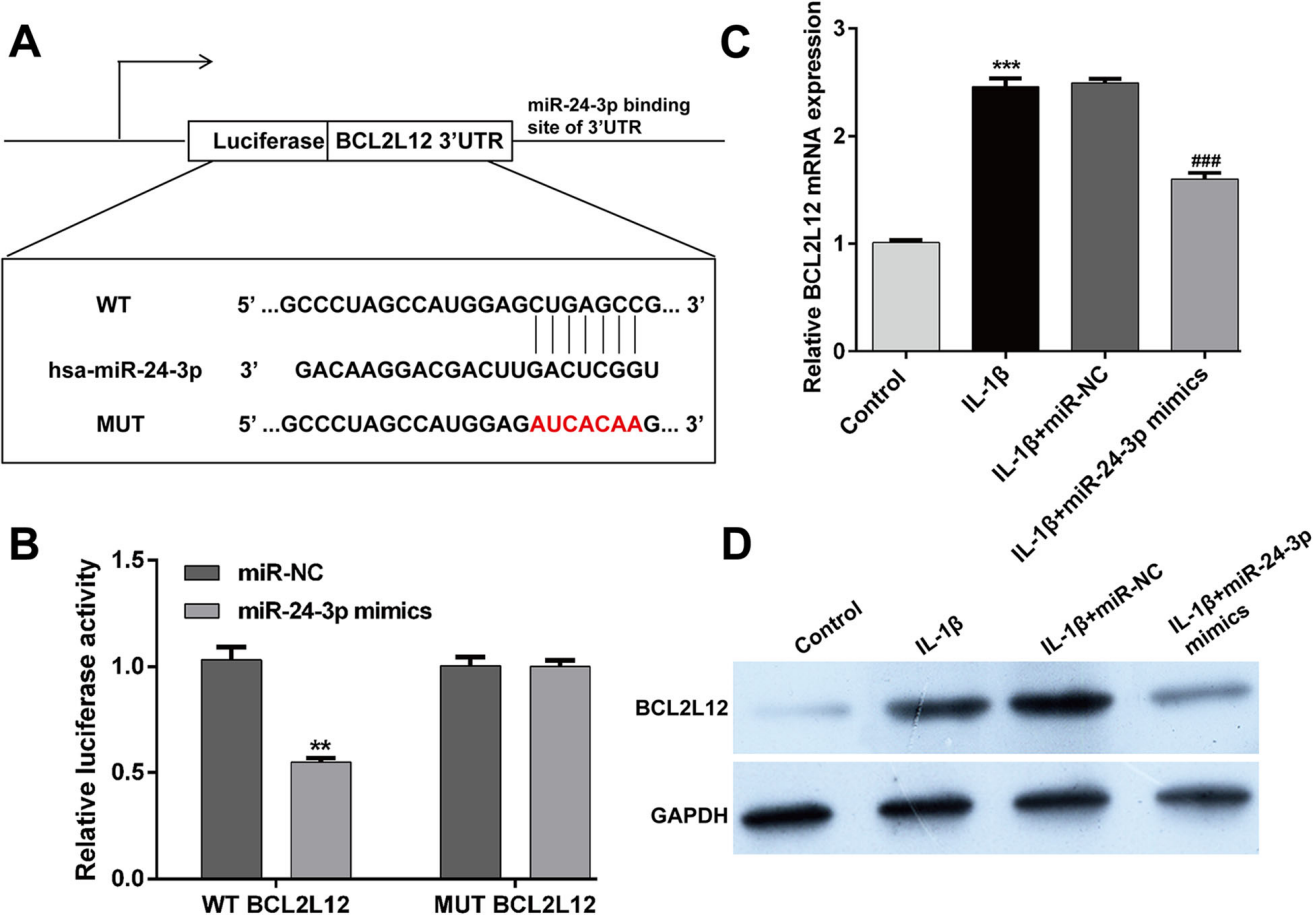
BCL2L12 was a direct target of miR-24-3p. **a** Alignment of the miR-24-3p seed sequence with the BCL2L12 3′ UTR (CUGAGCC). **b** The luciferase activity was measured in CHON-001 cells following co-transfecting with WT/MUT BCL2L12 3′-UTR plasmid and miR-24-3p with the luciferase reporter assay. **p < 0.01, compared with miR-NC; Chondrocyte cell line CHON-001 was transfected with miR-24-3p mimics or miR-NC, followed by IL-1β stimulation. The untreated cells were used as control group. **c** Expression level of BCL2L12 mRNA was determined via reverse transcription quantitative PCR. Data were presented as mean ± SD of three independent experiments. ***p < 0.001, compared with control; ###*p* < 0.001, compared with IL-1β + miR-NC; **d** Expression level of BCL2L12 protein in CHON-001 cells was detected with Western blotting

Next, we performed bioinformatics perdition to identify the putative targets of miR-24-3p by using TargetScan 7.1. Among the predicted targets, BCL2L12 was reported to be associated with OA pathogenesis, which thus was selected as a potential target of miR-24-3p. As shown in Fig. 3a, miR-24-3p and its binding sites in the 3′-UTR of BCL2L12 are highly conserved. To validate their interaction, luciferase reporter assay was performed in CHON-001 cells. As illustrated in Fig. 3b, co-transfection of miR-24-3p and BCL212 3′-UTR luciferase reporter plasmids significantly reduced the luciferase activity, whereas a mutated BCL2L12 3′UTR sequence prevented this reduction. To further confirm that BCL2L12 was negatively regulated by miR-24-3p, the mRNA and protein expression levels of BCL2L12 were analyzed by reverse transcription quantitative PCR and western blot analyses. We found that miR-24-3p mimics transfection significantly suppressed the expression of BCL2L12 at the mRNA (Fig. 3c) and protein (Fig. 3d) levels in IL-1β-stimulated CHON-001 cells. These data suggested that BCL2L12 might be a direct target of miR-24-3p.

### Knockdown of BCL2L12 imitated the protective effects of miR-24-3p against IL-1β-induced chondrocyte injury in vitro

**Fig. 4:**
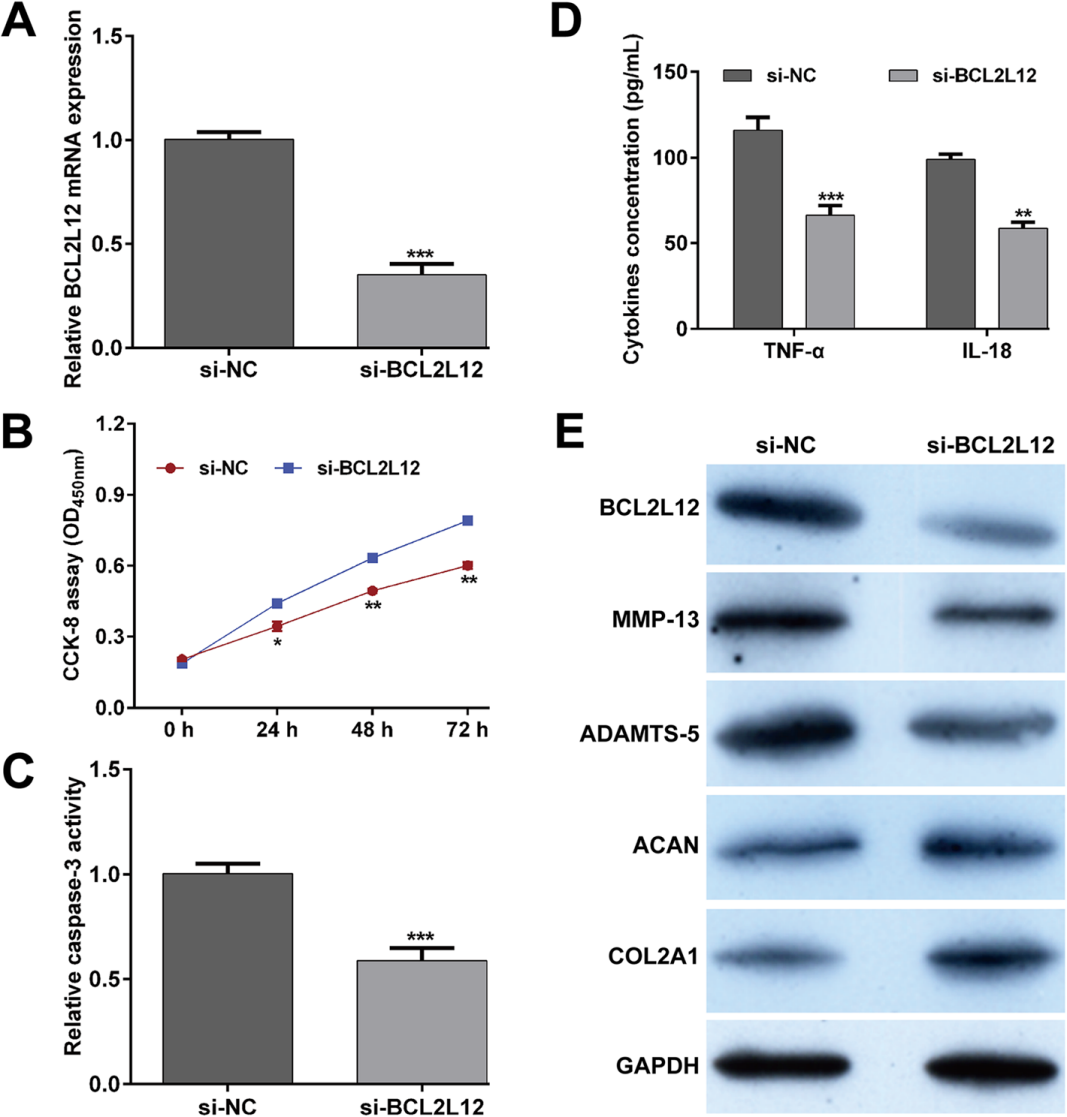
Effects of BCL2L12 knockdown on IL-1β-induced apoptosis, inflammation, and cartilage ECM degradation. Chondrocyte cell line CHON-001 was transfected with si-BCL2L12 or si-NC, followed by IL-1β stimulation. **a** The expression of BCL2L12 was detected by reverse transcription quantitative PCR. **b** Cell viability was examined by CCK-8 assay. **c** Caspase-3 activity was analyzed using by commercial Caspase-3 Colorimetric Activity Assay Kit. **d** The release of TNF-α and IL-18 in supernatant of CHON-001 cells from different groups was determined by ELISA assay. Data were presented as mean ± SD of three independent experiments. *p < 0.05, **p < 0.01, ***p < 0.001, compared with si-NC; **e** The protein levels of BCL2L12, MMP-13, ADAMTS-5, ACAN, and COL2A1 were measured by Western blot assay

As BCL2L12 as a target of miR-24-3p was upregulated in IL-1β-stimulated CHON-001 cells, we then transfected si-BCL2L12 or si-NC into CHONO-001 cells under IL-1β stimulation to investigate the possible effects of BCL2L12 on IL-1β-induced chondrocyte injury in vitro. The data of western blot analysis showed that the expression of BCL2L12 protein was obviously downregulated after si-BCL2L12 transfection in IL-1β-stimulated CHON-001 cells (Fig. 4a). Using constructed BCL2L12 silenced cell model, we performed a series of functional assays using CCK-8 assay, caspase-3 activity assay, ELISA assay, and western blot analysis. Our data indicated that downregulation of BCL2L12 reversed the repression of cell viability (Fig. 4b) and elevation of caspase-3 activity (Fig. 4c) mediated by IL-1β in CHON-001 cells. Additionally, increased concentration of pro-inflammatory cytokines (TNF-α and IL-18) in IL-1β-stimulated CHON-001 cells was attenuated after BCL2L12 knockdown (Fig. 4d). In IL-1β-stimulated CHON-001 cells, we also found that knockdown of BCL2L12 downregulated the protein expression of BCL2L12, MMP-13, and ADAMTS-5, while upregulated the protein expression of ACAN and COL2A1 (Fig. 4e).

### BCL2L12 participated in the miR-24-3p-induced protective effects against IL-1β-induced chondrocyte injury in vitro

**Fig. 5:**
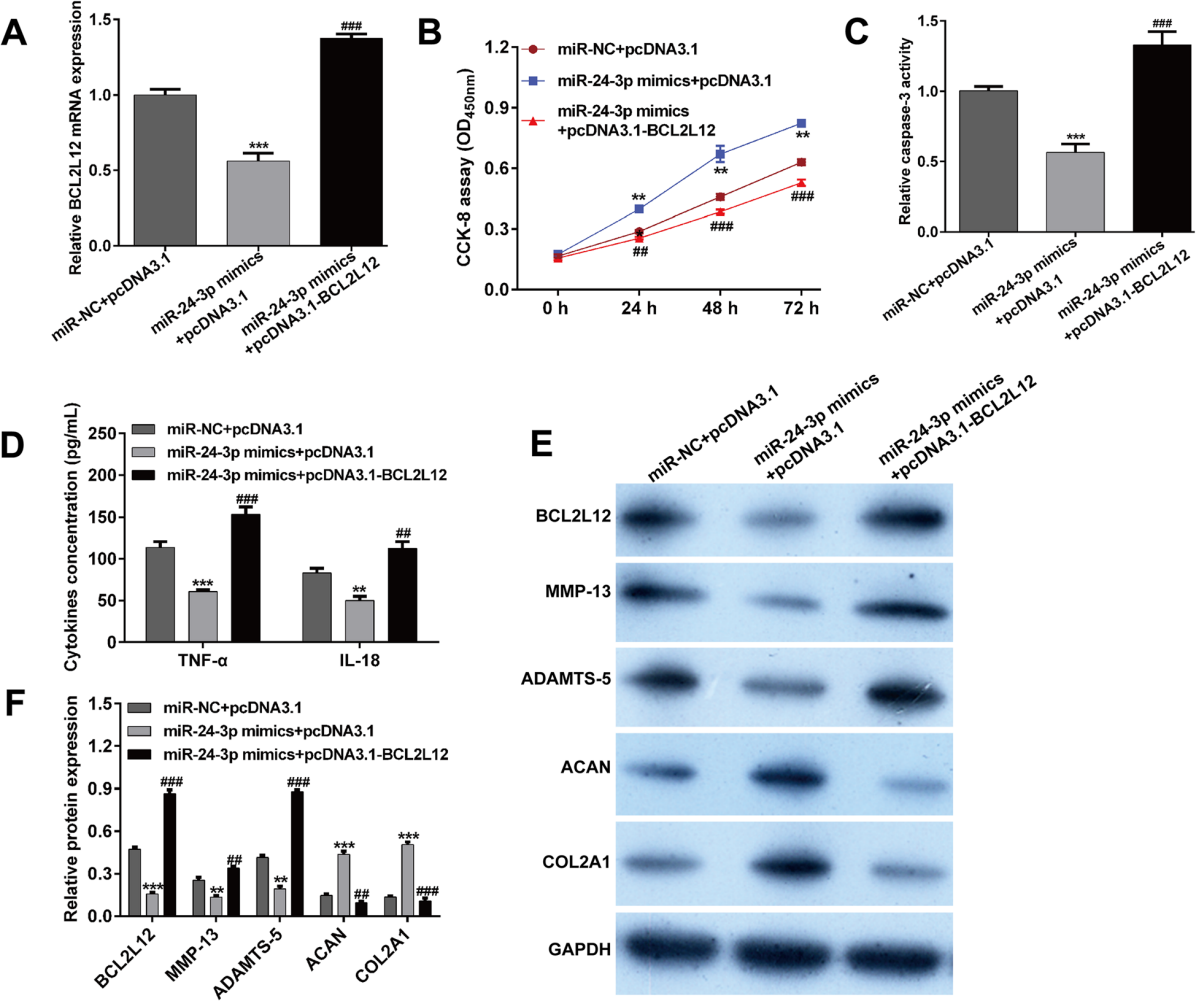
BCL2L12 participated in the miR-24-3p-induced protective effects against IL-1β-induced chondrocyte injury in vitro. Chondrocyte cell line CHON-001 was co-transfected with miR-24-3p mimics and pcDNA3.1-BCL2L12 or pcDNA3.1, followed by IL-1β stimulation for 24 h. **a** The expression of BCL2L12 was detected by reverse transcription quantitative PCR. **b** Cell viability was examined by CCK-8 assay. **c** Caspase-3 activity was analyzed using by commercial Caspase-3 Colorimetric Activity Assay Kit. **d** The release of TNF-α and IL-18 in supernatant of CHON-001 cells from different groups was determined by ELISA assay. **e**, **f** The protein levels of BCL2L12, MMP-13, ADAMTS-5, ACAN, and COL2A1 were measured by Western blot assay. Data were presented as mean ± SD of three independent experiments. **p < 0.01, ***p < 0.001, compared with miR-NC + pcDNA3.1; ##*p* < 0.01, ###*p* < 0.001, compared with miR-24-3p mimics + pcDNA3.1

As miR-24-3p could suppress the IL-1β-induced chondrocyte injury and negatively regulated BCL2L12 expression, we thus speculated that BCL2L12 might be the downstream regulator involved in miR-24-3p exerting functions. To test this, pcDNA3.1-BCL2L12 was transfected into IL-1β-stimulated CHON-001 cells in the presence of miR-24-3p mimics. After cultured for 48 h, we first found that decreased expression of BCL2L12 mRNA in miR-24-3p mimics transfected cells was reversed after pcDNA3.1-BCL2L12 transfection (Fig. 5a). Subsequently, the in vitro functional experiments demonstrated that miR-24-3p overexpression led to obvious increase in cell viability (Fig. 5b) and distinct decrease in caspase-3 activity (Fig. 5c), inflammation (Fig. 5d), and cartilage ECM degradation (Fig. 5e–f) in IL-1β-stimulated chondrocytes, while these effects were all ameliorated by BCL2L12 overexpression. Taken together, miR-24-3p significantly inhibited IL-1β-induced chondrocyte injury in vitro might via suppression of BCL2L12.

## Discussion

Investigation on the functional role of pivotal miRNAs associated with pathogenesis of OA may assist to in developing potential therapeutic strategies for OA patients. Here, we first found that the expression of miR-24-3p was significantly downregulated in OA cartilage tissues compared with normal cartilage tissues as well as IL-1β-stimulated CHON-001 cells compared to the control group. In fact, CHON-001 cells are the only components in healthy cartilage and mainly participate in maintaining and producing new cartilaginous matrix, whose apoptosis was positively associated with cartilage destruction in patients with OA [23, 24]. Higher levels of inflammatory cytokines, such as interleukin (IL)-1β and tumor necrosis factor (TNF)-α are frequently found in OA patients [25]. Accumulating evidence has indicated that IL-1β-stimulated CHON-001 cells could be used as OA model in vitro [26–28]. Therefore, it was appropriate to used IL-1β-stimulated CHON-001 cell model to investigate the functional role of miR-24-3p on inflammation and apoptosis involved in the pathogenesis of OA.

Functionally, we further demonstrated that overexpression of miR-24-3p remarkedly IL-1β-induced inflammation, caspase-3 activity, and cartilage ECM degradation in chondrocytes. Consistent with our data, miR-24-3p has been reported to exert protective effects against ischemia/reperfusion (I/R) injury [14, 15] and hepatic I/R process [16]. On the contrary, miR-24-3p upregulation could promote intervertebral disc degeneration through IGFBP5 and the ERK signaling pathway [29]. According to the report by Ragni et al. [17] who showed a strong capacity for adipose-derived MSCs (ASCs) to reduce matrix degradation activities, we thus inferred that miR-24-3p suppressed OA progression might via inhibiting apoptosis, inflammation, and ECM degradation.

In molecular levels, we further demonstrated that miR-24-3p overexpression weakened IL-1β-induced cartilage degradation, as reflected by reduction of MMP-13 and ADAMTS-5 protein expression, as well as markedly elevation of COL2A1 and ACAN protein expression in IL-1β-stimulated CHON-001 cells. As our best knowledge, the ECM is an important structure for maintaining the internal stability and structural integrity of cartilage and protecting the ECM from degeneration is one way to maintain chondrocyte function. The upregulation of MMP and ADAMTS production and downregulation of collagen and proteoglycan levels are correlated with the increases in apoptotic cells and ECM degradation in OA, which lead to matrix degradation [30]. In the other hand, chondrocyte apoptosis and inflammation are known to associate with the risk of cartilage loss and progression, as well as the clinical characteristics of OA [31, 32]. Under inflammatory conditions, including IL-1β stimulation, chondrocytes, as the only cell type residing in the cartilage, participate in the catabolic activities that ultimately cause the degradation of cartilaginous ECM [33]. In this study, miR-24-3p mimics inhibited the production of pro-inflammatory cytokines (TNF-α and IL-18) and enhanced matrix protein expression (COL2A1 and ACAN) while suppressing the levels of catabolic factors (MMP13 and ADAMTS-5), suggesting that miR-24-3p reduced inflammation and cartilage ECM degradation.

Furthermore, we performed luciferase reporter analysis to confirm that BCL2L12 was a direct target gene of miR-24-3p. In IL-1β-stimulated chondrocytes, the expression of BCL2L12 was significantly upregulated, which was notably decreased after miR-24-3p overexpression. We further demonstrated that BCL2L12 knockdown imitated, while overexpression significantly abrogated the protective effects of miR-24-3p against IL-1β-induced apoptosis, inflammation, and cartilage ECM degradation. Similar to the pro-apoptotic of BCL2L12 in IL-1β-stimulated chondrocytes, BCL2L12 participated in the induction of aberrant Th2-biased inflammation in the intestinal mucosa [34] and chronic rhinosinusitis [35] with allergy. Additionally, BCL2L12 exerts pro-apoptotic effects implicated in various malignancies, including laryngeal squamous cell carcinoma [36], breast cancer [37], and acute myeloid leukemia [38]. Based on these evidences, we thus concluded that miR-24-3p plays a pivotal role in the pathogenesis of OA though directly targeting on BCL2L12.

## Conclusions

In summary, our data indicated that miR-24-3p expression level was lower in the OA cases than in the control patients and IL-1β decreased the expression of miR-24-3p in the chondrocytes. Overexpression of miR-24-3p suppressed apoptosis, inflammation, and ECM degradation in IL-1β-stimulated chondrocytes by targeting BCL2L12. These preliminary data might provide important insight into targeting miR-24-3p/BCL2L12 axis for developing potential therapeutic strategies for OA patients.
